# Correlates of Fetal Aortic Isthmus Diameter in Fetuses Considered Structurally Normal on Second-Trimester Assessment

**DOI:** 10.3390/jcm15207798

**Published:** 2026-10-09

**Authors:** Hasan Beyhekim, Fatma Beyhekim, Zekiye Soykan Sert, Elif Betül Esmer, Mete Bertizlioğlu, Görkem Aktaş, Gökçen Örgül, Aybike Tazegül Pekin

**Affiliations:** 1 Division of Perinatology, Department of Obstetrics and Gynecology, Faculty of Medicine, Selçuk University—Alaeddin Keykubat Campus, 42130 Konya, Türkiye; 2 Department of Obstetrics and Gynecology, Faculty of Medicine, Selçuk University—Alaeddin Keykubat Campus, 42130 Konya, Türkiye

**Keywords:** fetal aortic isthmus, fetal echocardiography, semilunar valves, thymus, hierarchical regression

## Abstract

**Background/Objectives:** Fetal aortic isthmus reference data have characterized its size as a function of gestational age; whether aortic and pulmonary valve diameters are associated with isthmic caliber in addition to gestational age has not been systematically examined. We examined the correlates of aortic isthmus diameter in fetuses considered structurally normal on second-trimester assessment. **Methods:** This retrospective single-center cohort study included 472 fetuses considered structurally normal on second-trimester assessment at 19 + 0–24 + 6 weeks. Aortic isthmus (AoI), aortic valve (AV), pulmonary valve (PV) and thymic transverse diameters were measured by a single operator. Three-step hierarchical linear regression modeled the AoI diameter (Model 1, gestational age; Model 2, +AV and PV; Model 3, +thymus), with variance inflation factors, regression diagnostics and internal validation conducted via repeated five-fold cross-validation (100 repeats). For sensitivity analyses, the gestational-age equivalents recorded in the clinical reports for femur length, biparietal diameter and head circumference were converted into total days and analyzed as continuous variables in place of the clinical gestational age. **Results:** AoI diameter was correlated with gestational age (r = 0.512), AV (r = 0.507), PV (r = 0.516) and thymic diameter (r = 0.397) (all *p* < 0.001). Gestational age alone accounted for 26.2% of the variance (R^2^ = 0.262). Adding AV and PV increased the explained variance (ΔR^2^ = 0.060; total R^2^ = 0.322; *p* < 0.001); both AV (β = 0.186; *p* = 0.005) and PV (β = 0.187; *p* = 0.006) were independently associated with isthmic caliber, and the highest VIF in Model 2 was 3.22. Adding thymic diameter produced no meaningful increment (ΔR^2^ = 0.0003; *p* = 0.636). Across the four size-index specifications, the valve-term increment ranged from 0.037 to 0.060. Internal validation via repeated five-fold cross-validation gave a mean cross-validated R^2^ of 0.299 (SD of repeat-specific means, 0.011). **Conclusions:** In fetuses considered structurally normal on second-trimester assessment, aortic isthmus diameter was associated with gestational age, and, in addition, with aortic and pulmonary valve diameters, which accounted for a modest further share of the variance. No independent association with thymic diameter was demonstrated.

## 1. Introduction

The aortic arch system develops from the paired pharyngeal arch arteries, which undergo extensive and asymmetric remodeling during embryogenesis to produce the definitive arch and its branches. Cardiac neural crest cells migrate into the third, fourth and sixth pharyngeal arches and into the cardiac outflow tract, where they participate in the development and remodeling of the aortic arch system [1]. In the fetus, the aortic isthmus occupies a distinctive hemodynamic position between the left subclavian artery and the ductal junction [2].

Its caliber and flow characteristics have attracted attention as potential markers of fetal cardiovascular adaptation, particularly in growth restriction and congenital heart disease [3].

Existing descriptions of the fetal aortic isthmus have concentrated largely on establishing reference ranges for its diameter and Doppler indices as a function of gestational age [4,5,6,7]. Such gestational-age-based references implicitly treat isthmic size as a passive function of chronological maturation. However, the isthmus lies in anatomical and hemodynamic continuity with the ventricular outflow tracts, and its dimensions may be related to the calibers of the aortic and pulmonary valves as well as to gestational age. Whether semilunar valve biometry is associated with variation in isthmic caliber in addition to that attributable to gestational age has not, to our knowledge, been systematically examined.

A further question concerns the fetal thymus. The thymus arises from the same pharyngeal region, and cardiac neural crest cells contribute to development of the pharyngeal arch and aortic arch system and also participate in thymic development [1]. Cardiovascular anomalies are a well-recognized feature of 22q11.2 deletion syndrome, in which cardiac and thymic structures may be affected together [8,9]. This developmental background provides a rationale for asking whether thymic size and aortic isthmus diameter covary in fetuses without structural abnormality. It does not, however, establish that a measurable biometric relationship should exist across the structurally normal range, and whether such a relationship exists has not, to our knowledge, been examined.

We therefore conducted a retrospective cohort study of fetuses considered structurally normal on second-trimester assessment to examine the correlates of aortic isthmus diameter. Using a hierarchical regression framework, we examined whether semilunar valve diameters (aortic and pulmonary valve) and thymic transverse diameter were each associated with isthmic caliber in addition to gestational age, and we examined the stability of these associations when alternative fetal-size indices were substituted for the clinical gestational age.

## 2. Materials and Methods

### 2.1. Study Design and Population

This retrospective cohort study was conducted at the Perinatology Division of Selçuk University Faculty of Medicine. The institutional fetal ultrasound archive was searched to identify singleton pregnancies that underwent detailed second-trimester fetal echocardiographic assessment between 1 January 2023 and 12 December 2025.

Gestational age was based on the last menstrual period, with first-trimester crown–rump length used for dating when there was a clinically relevant discrepancy between the menstrual and sonographic estimates.

Inclusion criteria were a singleton, viable pregnancy; a fetus without structural abnormality identified at the index second-trimester examination and without known chromosomal abnormality; a gestational age between 19 + 0 and 24 + 6 weeks, determined as described above; and complete measurements of the aortic isthmus, aortic valve, pulmonary valve and thymus. Exclusion criteria were multiple gestation; major structural anomaly; abnormal genetic screening or confirmed chromosomal anomaly; intrauterine growth restriction; incomplete biometric data; and gestational age outside the predefined window.

Fetal growth was assessed clinically at the index examination, and estimated fetal weight was calculated using the Hadlock formula. Fetuses with a clinically established and documented diagnosis of fetal growth restriction at the index examination were excluded (*n* = 5); no research-specific retrospective screening of estimated-fetal-weight percentiles was performed when the cohort was assembled. Applied retrospectively to the deposited dataset, the Hadlock in utero weight standard [10] classifies 9 of the 468 retained fetuses with a recorded estimated fetal weight as below the 10th percentile for gestational age; these fetuses had not been clinically diagnosed and documented as growth-restricted at the index examination, and were therefore retained.

**Figure 1:**
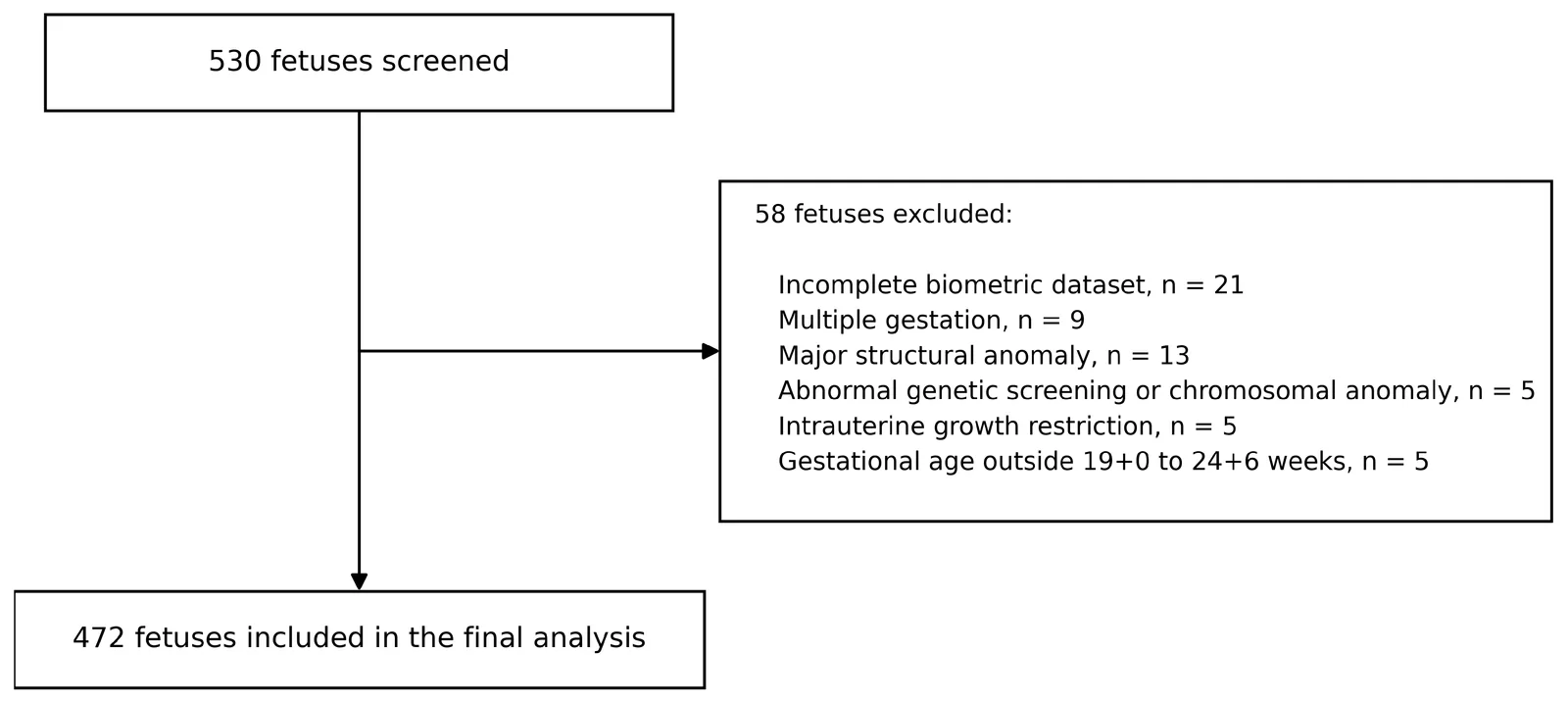
Flow diagram of study cohort selection.

Of the 530 fetuses screened, 58 were excluded (incomplete biometric dataset, 21; multiple gestation, 9; major structural anomaly, 13; abnormal genetic screening or chromosomal anomaly, 5; intrauterine growth restriction, 5; and gestational age outside 19 + 0 to 24 + 6, 5), yielding a final cohort of 472 fetuses (Figure 1).

Ascertainment of normality was limited to the index second-trimester examination. The institutional archive does not contain a systematic postnatal cardiac verification variable, and no systematic postnatal echocardiographic assessment was performed for the cohort. A reliable variable recording the findings of subsequent prenatal scans was likewise not available. Consequently, cardiac lesions that were not apparent at the index second-trimester assessment—including coarctation of the aorta, which characteristically evolves and may only become manifest in the third trimester or after birth—could have been present and unrecognized. The cohort is therefore described throughout as structurally normal on second-trimester assessment, and no claim of confirmed postnatal normality is made.

No a priori sample-size calculation was performed; the study sample comprised all examinations meeting the predefined eligibility criteria during the study period.

### 2.2. Echocardiographic Measurements

All examinations were performed transabdominally by a single experienced operator using a Voluson E6 system (GE Healthcare, Milwaukee, WI, USA) equipped with a C2-9-D convex transducer. All measurements were obtained prospectively during the clinical examination and were subsequently retrieved from the institutional archive for the present analysis.

Cardiac and vascular diameters (aortic isthmus, aortic valve and pulmonary valve) were measured using the inner-to-inner technique during systole, when the respective valve or vessel reached its maximum dimension. The aortic isthmus diameter was measured in the long-axis view of the aortic arch, at its narrowest segment between the origin of the left subclavian artery and the junction with the ductus arteriosus, perpendicular to the vessel axis. The aortic valve diameter was measured at the level of the valve leaflets in the left ventricular outflow tract view, and the pulmonary valve diameter at the level of the valve leaflets in the right ventricular outflow tract view. The transverse diameter of the thymus was measured separately as its widest transverse dimension in the three-vessel–trachea view.

The archive holds the clinical reports and their stored numerical measurements; images and clips suitable for blinded offline re-measurement were not available. Retrospective repeat measurement was therefore not possible, intraobserver and interobserver reproducibility could not be assessed, and no intraclass correlation coefficient or repeatability coefficient is reported.

### 2.3. Fetal Size Indices

The clinical reports recorded gestational-age equivalents corresponding to biparietal diameter, head circumference and femur length in weeks and days (for example, “22W4D”). These variables are referred to below as the BPD-derived, HC-derived and FL-derived ages.

For the sensitivity analyses described below, each age equivalent was converted into total days and analyzed as a continuous variable. These analyses were used to assess the stability of the findings when alternative biometric-derived age indices were substituted for the clinical gestational age. No formal statistical comparison between the alternative indices was performed, and the resulting R^2^ values were not used to rank them.

### 2.4. Statistical Analysis

Statistical analyses were performed using Python (Google Colab) with the pandas, scipy, statsmodels and scikit-learn libraries; the reported statistical analyses were reproduced with Python 3.13.16 (pandas 2.2.3, numpy 2.1.3, scipy 1.16.3, statsmodels 0.15.0 and scikit-learn 1.6.1). Gestational age was treated as a continuous variable (completed weeks plus days divided by seven). Continuous variables are summarized as mean ± standard deviation. Pearson correlation coefficients were calculated between the aortic isthmus diameter and each candidate correlate.

All analyses were performed on available cases without imputation. The aortic isthmus, aortic valve, pulmonary valve and thymic diameters and all derived gestational-age equivalents were complete for all 472 fetuses, so the hierarchical models, the ratio distributions and the reference equations were based on the full cohort. Estimated fetal weight was unavailable in four fetuses and is reported descriptively for the remaining 468. Fetal sex was indeterminate in 58 fetuses, and the sensitivity analysis including sex was therefore performed in the 414 fetuses with a single unambiguous recorded sex.

The associations of gestational age, semilunar valve diameters and thymic size with aortic isthmus diameter were assessed using three-step hierarchical linear regression. Model 1 included gestational age; Model 2 added the aortic and pulmonary valve diameters; and Model 3 added the thymic transverse diameter. The change in the coefficient of determination (ΔR^2^) and the associated F-test were evaluated at each step, and standardized β coefficients were derived for each model. Multicollinearity was assessed using variance inflation factors. Regression assumptions were examined by inspection of residuals, the Shapiro–Wilk test of residual normality, the Breusch–Pagan test of homoscedasticity, and Cook’s distance, with observations exceeding the conventional 4/*n* threshold enumerated.

Internal validation of Model 2 was performed by repeated k-fold cross-validation: 5 folds repeated 100 times, giving 500 validation folds, with the random seed fixed at 42 so that the result would be exactly reproducible from the deposited script. The mean cross-validated R^2^ and the standard deviation of the repeat-specific mean R^2^ are reported. The seed was fixed for reproducibility and was not prespecified before the analysis.

The entire hierarchical sequence was repeated three times more, substituting in turn the biparietal-diameter-derived, head-circumference-derived and femur-length-derived gestational-age equivalents for the clinical gestational age, in order to examine whether the valve and thymic findings were dependent on the particular size index chosen.

Isthmus-to-valve ratios (aortic isthmus divided by aortic valve, and by pulmonary valve) were computed for each fetus and summarized by completed gestational week, with empirical 5th and 95th percentiles. These ratios are reported as descriptive summaries; they are not presented as inherently size-independent quantities, and their residual association with the size indices is reported.

Descriptive equations for aortic isthmus diameter were derived on gestational age and, separately, on the femur-length-derived gestational-age equivalent. The conditional mean was modeled by ordinary least squares. The conditional standard deviation was modeled as a function of the predictor using the absolute-residual method: absolute residuals from the mean model were scaled by √(π/2) and regressed on the predictor by iteratively weighted least squares with weights 1/σ^2^, following Altman [11], in the form widely applied to fetal reference data [12]. A quadratic mean term in gestational age was examined but not retained (Section 3.7).

Fetal sex was recorded as a single unambiguous value in 414 fetuses (200 female and 214 male); of the remaining 58, 56 had no sex entry and two contained conflicting entries, and all were treated as indeterminate. A sensitivity analysis adding fetal sex as a covariate to each of the reported models was performed in these 414 fetuses; sex was not included in the primary models.

A two-sided *p* value < 0.05 was considered statistically significant.

## 3. Results

### 3.1. Cohort Characteristics

A total of 530 fetuses were screened from the institutional archive. After exclusion of 58 cases (incomplete biometric dataset, 21; multiple gestation, 9; major structural anomaly, 13; abnormal genetic screening or chromosomal anomaly, 5; intrauterine growth restriction, 5; and gestational age outside the 19 + 0 to 24 + 6 window, 5), the final cohort comprised 472 fetuses considered structurally normal on second-trimester assessment (Figure 1).

Maternal age was 28.6 ± 4.7 years, and mean gestational age was 21.6 ± 1.2 weeks (range, 19.0–24.9; i.e., 19 + 0 to 24 + 6). Fetal sex was determinate in 414 cases (female, 200 [48.3%]; male, 214 [51.7%]) and indeterminate in 58. Estimated fetal weight was recorded in 468 fetuses and averaged 477.0 ± 120.8 g.

The mean measurements were as follows: aortic isthmus, 2.05 ± 0.38 mm; aortic valve, 3.00 ± 0.51 mm; pulmonary valve, 3.82 ± 0.66 mm; and thymic transverse diameter, 13.58 ± 2.39 mm. The ratio of mean pulmonary to mean aortic valve diameter was 1.27, consistent with the expected right-ventricular predominance of the second-trimester fetal circulation (Table 1).

Mean diameters increased across gestational weeks. Between 19 and 24 weeks, the aortic isthmus increased from 1.78 ± 0.17 to 2.67 ± 0.36 mm, the aortic valve from 2.41 ± 0.27 to 3.91 ± 0.68 mm, the pulmonary valve from 3.04 ± 0.33 to 5.06 ± 0.82 mm, and the thymus from 10.67 ± 1.21 to 17.91 ± 2.50 mm (Table 2).

Aortic isthmus diameter was correlated with gestational age (r = 0.512), aortic valve diameter (r = 0.507), pulmonary valve diameter (r = 0.516) and thymic transverse diameter (r = 0.397) (all *p* < 0.001; Table 3).

### 3.2. Hierarchical Regression

Results of the hierarchical linear regression are shown in Table 4. In Model 1, gestational age alone accounted for 26.2% of the variance in aortic isthmus diameter (R^2^ = 0.262; F = 167.1; *p* < 0.001). Adding the semilunar valve diameters in Model 2 produced a significant increment in explained variance (ΔR^2^ = 0.060; F-change = 20.73; *p* < 0.001), raising the total to 32.2% (R^2^ = 0.322); within this model, both aortic valve (β = 0.186; *p* = 0.005) and pulmonary valve (β = 0.187; *p* = 0.006) diameters were independently associated with isthmic caliber, and gestational age retained the largest standardized coefficient (β = 0.256; *p* < 0.001). The highest variance inflation factor in Model 2 was 3.22. Adding the thymic transverse diameter in Model 3 produced no meaningful improvement in model fit (ΔR^2^ = 0.0003; F-change = 0.22; *p* = 0.636), and thymic size was not independently associated with aortic isthmus diameter (β = 0.025; *p* = 0.636). In Model 3, gestational age (β = 0.245; *p* < 0.001), aortic valve (β = 0.183; *p* = 0.006) and pulmonary valve (β = 0.183; *p* = 0.008) each remained associated with the isthmus.

### 3.3. Sensitivity to the Choice of Size Index

The hierarchical sequence was repeated substituting each biometric-derived age for the clinical gestational age (Table 5). The increment in explained variance attributable to the semilunar valve terms ranged from 0.037 to 0.060 across the four specifications (ΔR^2^ = 0.060 for gestational age, 0.050 for the FL-derived age, 0.053 for the BPD-derived age and 0.037 for the HC-derived age), and both valve terms remained statistically significant in every specification. The thymic term remained non-significant in all four (*p* = 0.636, 0.916, 0.946 and 0.730, respectively).

No formal statistical comparison between the four indices was performed, so the R^2^ values in Table 5 do not rank them, and none of these analyses supports a claim that the valve associations are independent of fetal size.

### 3.4. Model Diagnostics and Internal Validation

Diagnostic evaluation of Model 3 showed no evidence of heteroscedasticity on the Breusch–Pagan test (*p* = 0.700). Residuals departed from normality (Shapiro–Wilk W = 0.979; *p* < 0.001). The maximum Cook’s distance was 0.029; 31 observations (6.6%) exceeded the 4/*n* criterion. Variance inflation factors ranged from 1.88 to 3.28.

Internal validation of Model 2 by repeated five-fold cross-validation (100 repeats; 500 validation folds; random seed fixed at 42 for reproducibility) gave a mean cross-validated R^2^ of 0.299, with a standard deviation of the repeat-specific mean R^2^ of 0.011. This is an internal estimate and does not constitute external validation.

### 3.5. Fetal Sex

Among the 414 fetuses with a single unambiguous recorded sex, aortic isthmus diameter was 2.012 ± 0.364 mm in females and 2.088 ± 0.408 mm in males; the unadjusted difference was of borderline statistical significance (*t* = 2.00; *p* = 0.047). When fetal sex was added to each of the four fully adjusted specifications, the sex term was not statistically significant in any of them (gestational-age model, B = +0.056 mm, *p* = 0.080; femur-length-derived model, B = +0.053 mm, *p* = 0.099; biparietal-diameter-derived model, B = +0.014 mm, *p* = 0.663; head-circumference-derived model, B = +0.023 mm, *p* = 0.469; B denotes the unstandardized coefficient, in millimeters), and the valve and thymic findings were unchanged. The small unadjusted difference was therefore attenuated after adjustment, and the sex term was not statistically significant in the adjusted models; this does not establish the absence of a sex effect, and the analysis was restricted to the 414 fetuses with a single unambiguous recorded sex.

### 3.6. Isthmus-to-Valve Ratios

Isthmus-to-valve ratios by completed gestational week are shown in Table 6. Across the whole cohort, the aortic isthmus measured, on average, 0.693 of the aortic valve diameter (5th–95th percentile, 0.515–0.920) and 0.544 of the pulmonary valve diameter (5th–95th percentile, 0.405–0.714).

These ratios are descriptive summaries and are not size-independent: both retained a small but statistically detectable negative association with gestational age (aortic isthmus/aortic valve, r = −0.112, *p* = 0.015; aortic isthmus/pulmonary valve, r = −0.146, *p* = 0.001), and similar small associations were present with the derived size indices. Ratios are therefore reported to characterize the proportionality of the three structures within this cohort, not as a means of removing the influence of fetal size.

### 3.7. Descriptive Equations for Aortic Isthmus Diameter

**Figure 2:**
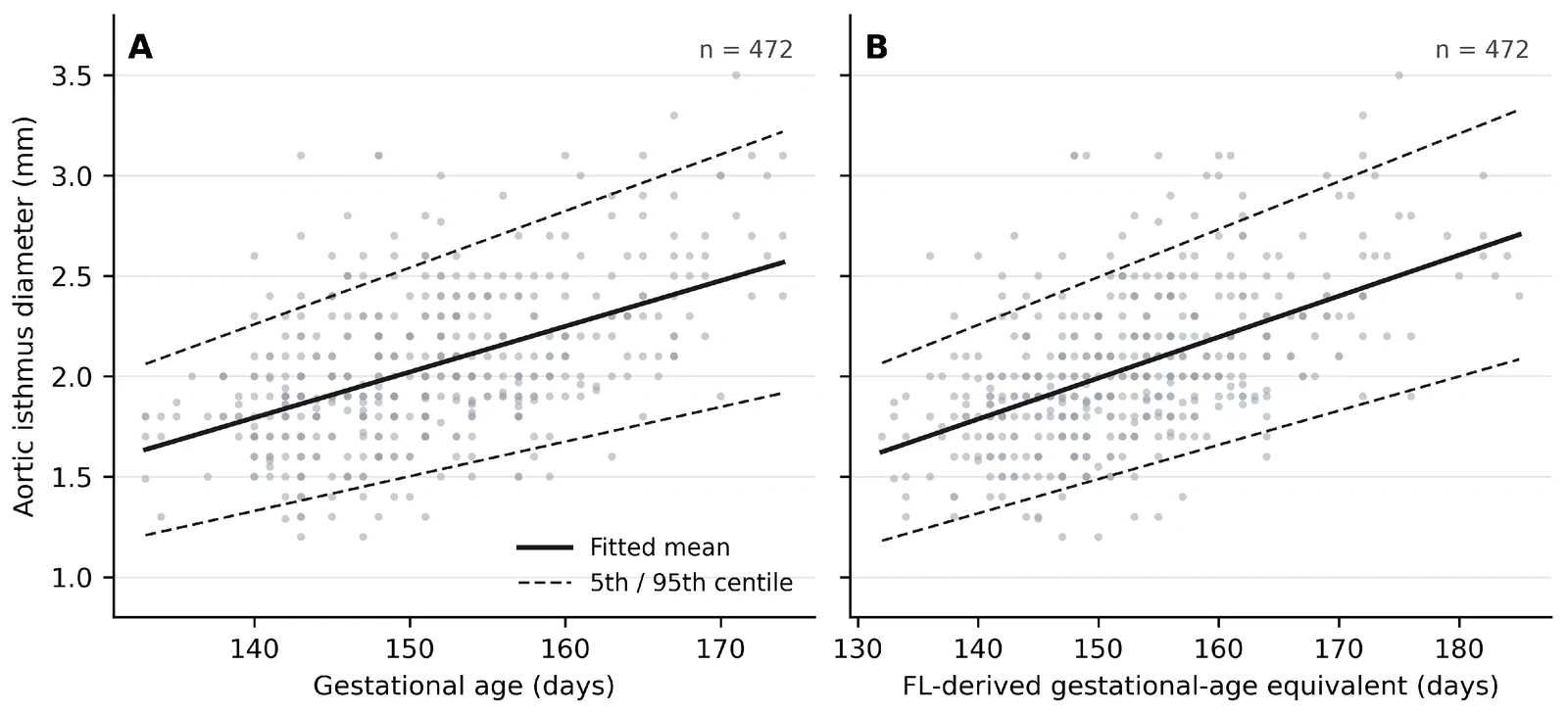
Aortic isthmus diameter in 472 fetuses considered structurally normal on second-trimester assessment, plotted against gestational age (panel (**A**)) and against the femur-length-derived gestational-age equivalent (panel (**B**)). The solid line is the fitted mean and the dashed lines are the fitted 5th and 95th centiles, calculated as mean(x) ± 1.645 × SD(x), where the mean was fitted by ordinary least squares and the standard deviation was obtained from the absolute residuals scaled by √(π/2) and modeled on the predictor by weighted least squares following Altman [11]. Panel (**B**) uses the FL-derived age in total days, as defined in Section 2.3. The fitted centiles are descriptive centiles for this cohort and apply within the observed ranges (133–174 days in panel (**A**) and 132–185 days in panel (**B**)); external validation was not performed. Light-gray points represent individual observations; darker points indicate overlapping values.

Descriptive equations relating aortic isthmus diameter to gestational age and, separately, to the femur-length-derived gestational-age equivalent are given in Table 7 and displayed in Figure 2. In each case, the conditional mean and the conditional standard deviation were both modeled as linear functions of the predictor, so that a standardized deviation for an individual measurement can be computed as z = (observed − predicted mean)/predicted SD.

A quadratic mean term in gestational age was examined and not retained, because its contribution was not robust to the composition of the upper end of the gestational-age window: removal of the 21 fetuses examined at 24 weeks changed the associated *p* value from 0.007 to 0.379.

The equations are descriptive and apply within the predictor ranges observed here—133 to 174 days for gestational age and 132 to 185 days for the FL-derived age. External validation was not performed.

## 4. Discussion

### 4.1. Principal Findings

In this cohort of 472 fetuses considered structurally normal on second-trimester assessment, aortic isthmus diameter was associated with gestational age and, in addition, with the diameters of the aortic and pulmonary valves. Gestational age had the largest standardized coefficient and alone accounted for 26.2% of the variance, while the two valve terms together added a statistically significant but modest 6.0 percentage points of explained variance (ΔR^2^ = 0.060; *p* < 0.001). When the FL-, BPD- and HC-derived ages were substituted for the clinical gestational age, the valve-term increment ranged from 0.037 to 0.053, compared with 0.060 in the gestational-age model. In contrast, thymic transverse diameter, although correlated with the isthmus in unadjusted analysis, showed no independent association once gestational age and semilunar valve dimensions were accounted for, and remained non-significant in all four model specifications.

### 4.2. Comparison with Previous Literature

Previous studies of the fetal aortic isthmus have largely characterized reference ranges for its diameter and Doppler indices across gestation, including pulsatility index reference ranges derived in both singleton and twin pregnancies [4,5,6,13]. Our approach differs in framing isthmic caliber as an outcome examined alongside the aortic and pulmonary valve diameters rather than against chronological age alone. The observed positive correlations between isthmic caliber and semilunar valve diameters are consistent with the anatomical and hemodynamic continuity between the ventricular outflow tracts and the aortic arch. The ratio of the mean pulmonary to mean aortic valve diameter (1.27) is in accordance with the physiological right-ventricular predominance of the human fetal circulation [14,15]. The thymic findings are considered in Section 4.4.

Age-matched comparison with published aortic isthmus data showed that our absolute measurements were systematically lower, with a weekly rate of increase close to that derived from the Udomwan medians and lower than that reported by Achiron et al. Across 19–24 weeks, our weekly mean values were approximately 0.51–0.75 mm lower than values calculated from the regression reported by Achiron et al. [7] and approximately 0.24–0.51 mm lower than the median values tabulated by Udomwan et al. [5]; at the mean gestational age of our cohort, the difference was approximately 0.64 mm for the Achiron comparison and approximately 0.43 mm for the Udomwan comparison. The weekly increase in our data (approximately 0.159 mm/week) was close to that derived from the tabulated Udomwan medians (approximately 0.154 mm/week) and lower than that reported by Achiron et al. (approximately 0.189 mm/week). Comparison with Pasquini et al. [6] was made using the gestational-age model published for the isthmus measured in the sagittal arch view, which corresponds to the plane used here (ln[predicted isthmal diameter] = 1.109 × ln[gestational age in weeks] − 2.489; *n* = 190). Values derived from that fitted model describe the conditional geometric mean on the logarithmic scale rather than observed means. Across 19–24 weeks, our weekly mean values were approximately 0.20–0.52 mm lower than the values derived from it, and approximately 0.46 mm (18.2%) lower at the mean gestational age of our cohort; the weekly increase derived from that model over the same interval (approximately 0.129 mm/week) was lower than in our data. Differences in absolute calibration may partly reflect methodological differences among studies, including imaging plane, timing within the cardiac cycle and caliper convention. Our measurements were obtained inner-to-inner during systole at maximum dimension at the narrowest segment of the aortic isthmus in the long-axis arch view. These methodological differences limit direct comparison of absolute values and we therefore do not interpret the lower measurements in our cohort as evidence of a biological difference. The gestational-age-specific values reported here, and the equations in Table 7 and the fitted centiles in Figure 2, accordingly reflect the measurement convention of this center.

### 4.3. Hemodynamic Interpretation

The observed association between semilunar valve dimensions and aortic isthmus diameter has a plausible physiological context. The aortic isthmus occupies a distinctive position within the parallel fetal circulation [2]. Fetal cardiac output is distributed unequally between the right and left ventricular circulations, with flow through the pulmonary and aortic outflow tracts contributing differently to the parallel fetal circulation [14,15]. Against this physiological background, the association observed here may reflect biological covariance, although these data cannot separate biological covariance from correlated examination-level measurement error (Section 4.7). The interpretation is therefore associative and hypothesis-generating, and the cross-sectional design cannot establish causal or directional hemodynamic relationships.

### 4.4. Thymic Findings

The thymus was examined because cardiac neural crest cells contribute to development of the pharyngeal arch and aortic arch system and also participate in thymic development [1], and because cardiac and thymic structures are coordinately affected in 22q11.2 deletion syndrome [8,9]. This background provided the rationale for the analysis rather than an expectation about its result.

Thymic diameter correlated with isthmic diameter in unadjusted analysis but we did not demonstrate an independent association after adjustment for the size index and semilunar valve dimensions. The thymic term was not statistically significant in any specification (*p* = 0.636, 0.916, 0.946 and 0.730 for the models indexed to gestational age and to the femur-length-, biparietal-diameter- and head-circumference-derived gestational-age equivalents respectively), and its addition produced essentially no incremental explanatory value. The apparent bivariate correlation with the thymus may largely reflect the shared dependence of both structures on gestational age.

These data therefore do not support the use of thymic diameter as a correlate or explanatory variable for aortic isthmus diameter in this cohort. A non-significant result in a cross-sectional biometric study is not evidence that no biological relationship exists, and the present design cannot adjudicate embryological mechanisms.

### 4.5. Indexing to Fetal Size

Because all measured structures enlarge with advancing gestation, a central question is whether the associations we observed reflect generalized fetal growth. Previous fetal echocardiographic studies have related cardiac dimensions to biometric measures of fetal size as well as to gestational age; Schneider et al. found femur length, used as an expression of fetal size, to have the highest correlation with fetal cardiac dimensions, while Pasquini et al. used femur length for normalization of aortic isthmus dimensions [6,16]; more recent reference ranges for fetal cardiac structures have likewise been modeled on gestational age together with biparietal diameter and femur length [17]. We therefore repeated the hierarchical models with gestational age replaced in turn by the femur-length-, biparietal-diameter- and head-circumference-derived gestational-age equivalents.

These substitutions use the FL-, BPD- and HC-derived ages defined in Section 2.3, which express the corresponding biometric parameters on an age scale. They are therefore sensitivity analyses of the choice of index rather than the millimeter-based fetal-size indexing that the literature recommends.

Two features were consistent across all four specifications. The aortic and pulmonary valve terms remained associated with isthmic diameter, and the thymic term remained non-significant. The incremental variance attributable to the valve terms was not stable across indices, ranging from 0.037 to 0.060; we report this range rather than a single value.

### 4.6. Interpretation and Future Directions

Aortic isthmus diameter in the second trimester varied with gestational age and, in addition, with the diameters of the aortic and pulmonary valves, by an amount that was statistically clear but modest. Gestational-age nomograms may therefore not capture all of the systematic variation in isthmic caliber. Whether aortic and pulmonary valve diameters provide clinically useful information beyond gestational age in the interpretation of an individual isthmic measurement requires further investigation. Outflow-tract diameters are measured prospectively in the assessment of suspected coarctation, although in that setting the parameters that distinguished confirmed from false-positive cases were arch and ductal measurements rather than the semilunar valve diameters themselves [18].

Accurate interpretation of the aortic isthmus is of clinical interest because reduced isthmic caliber is among the sonographic signs used in the prenatal assessment of aortic coarctation, a lesion for which antenatal diagnosis remains difficult [19,20]; a recent individual participant data meta-analysis found the diagnostic accuracy of prenatal ultrasound for coarctation to be only moderate even when combinations of parameters were considered [21]. The present cohort was not assembled to address coarctation and included no postnatal outcome verification, so these data describe the biometry of the isthmus rather than its diagnostic performance.

The findings indicate what a prospective study would need to include. Measurement reproducibility should be quantified rather than assumed; biometric parameters should be recorded as linear dimensions so that conventional fetal-size indexing can be applied; the gestational-age window should be widened; and postnatal cardiac outcome should be ascertained systematically so that the relationship between second-trimester isthmic biometry and later findings can be examined directly. Until such data exist, the present results are best read as a description of how these structures covary within one second-trimester cohort.

### 4.7. Strengths and Limitations

The strengths of this study include its moderately large sample and the fact that all examinations were performed by a single experienced operator. Single-operator measurement is not, however, a substitute for reproducibility assessment, which could not be performed here (see below). The analytic approach incorporated hierarchical modeling, variance inflation factor assessment, formal regression diagnostics, internal validation by repeated cross-validation with a fixed seed, and replication of the entire model sequence under alternative size indices. The de-identified analytic dataset and the analysis scripts are deposited as Supplementary Material (Supplementary Dataset S1 and Supplementary Files S1 and S2).

Several limitations should be acknowledged, of which the first is the most consequential.

First, no reproducibility data are available. Because the archive holds the clinical reports and their stored numerical measurements rather than images suitable for blinded offline re-measurement, neither intraobserver nor interobserver reproducibility could be assessed, and no intraclass correlation coefficient or repeatability coefficient is reported. Measurements were also obtained during routine clinical examinations rather than by standardized offline re-measurement, with the variability inherent to clinical practice. The measurement error attaching to each of the four diameters is therefore unquantified, and the magnitude of the incremental variance attributable to the valve terms cannot be interpreted with confidence. This also raises a specific alternative explanation for part of the central finding: the aortic isthmus, aortic valve and pulmonary valve were all measured by the same operator, in the same sitting, on the same fetus, often in adjacent views. Measurement error under those conditions is unlikely to be independent across structures—a scan in which the operator obtains slightly generous calipers on one outflow tract may well produce slightly generous calipers on the other, and common image quality acts on all of the measurements together. Correlated examination-level measurement error of this kind would inflate the apparent covariance between the isthmus and the semilunar valves and cannot be separated from genuine biological covariance in these data, so some part of the observed ΔR^2^ = 0.060 may be attributable to shared measurement error rather than to shared biology.

Second, ascertainment of normality was limited to the index second-trimester examination. There was no systematic postnatal cardiac verification and no systematic postnatal echocardiographic assessment, and no reliable record of subsequent prenatal scan findings was available. Lesions not apparent at the index assessment—coarctation of the aorta in particular, which frequently becomes manifest only later—could have been present and unrecognized. The cohort is accordingly described as structurally normal on second-trimester assessment, and confirmed postnatal normality is not claimed.

Third, the study was retrospective and conducted at a single center, which limits generalizability; external, multicenter validation is warranted. Because inclusion required complete measurements of the aortic isthmus, aortic valve, pulmonary valve and thymus, and 21 examinations were excluded for incomplete biometric data, the analytic cohort may preferentially represent examinations in which these structures could be successfully visualized and measured; this selection may limit generalizability. Because the absolute values obtained here are systematically lower than those of the published series with which they were compared, the equations in Table 7 and the fitted centiles in Figure 2 should not be used to classify an individual fetus, nor compared or interchanged with Z-scores derived from other references, without external validation.

Fourth, the biometric parameters were available only as the derived ages defined in Section 2.3, so conventional fetal-size indexing could not be performed; the sensitivity analyses address the choice of index but do not substitute for it, and no claim of independence from fetal size is made.

Fifth, the gestational-age window was narrow (19–24 weeks), with few observations at its extremes (*n* = 22 at 19 weeks and *n* = 21 at 24 weeks), which limits precision at the margins; this is also why a quadratic mean term was not retained in the descriptive equations.

Sixth, the incremental variance explained by semilunar valve biometry, although statistically significant in each specification, was modest in magnitude and ranged from 0.037 to 0.060 across the four specifications.

Seventh, fetal sex was available as a single unambiguous recorded value in 414 of 472 fetuses, so the sex sensitivity analysis was based on a subset; the sex term was not statistically significant after adjustment in any specification, but the absence of a sex effect was not established.

Finally, postnatal outcome data were not available, so the prognostic relevance of these associations remains undetermined.

## 5. Conclusions

In fetuses considered structurally normal on second-trimester assessment, aortic isthmus diameter was associated with gestational age and with aortic and pulmonary valve diameters. The valve terms provided a modest additional contribution to the explained variance beyond gestational age. We did not demonstrate an independent association between thymic diameter and aortic isthmus diameter in any model specification. Prospective, multicenter studies incorporating reproducibility assessment and postnatal outcomes are needed to establish the clinical relevance of these observations.

## Figures and Tables

**Table 1:** Demographic characteristics and overall biometric measurements of the study cohort (*n* = 472).

Characteristic	Value
Maternal age, years	28.6 ± 4.7
Gestational age, weeks	21.6 ± 1.2
Gravidity, median (IQR)	2 (1–2)
Parity, median (IQR)	0 (0–1)
Female fetus, *n* (%)	200 (48.3)
Male fetus, *n* (%)	214 (51.7)
Estimated fetal weight, g	477.0 ± 120.8
Aortic isthmus, mm	2.05 ± 0.38
Aortic valve, mm	3.00 ± 0.51
Pulmonary valve, mm	3.82 ± 0.66
Thymus transverse diameter, mm	13.58 ± 2.39
Pulmonary/aortic valve ratio	1.27

Values are mean ± standard deviation unless otherwise stated. Denominators differ between variables where data were incomplete: gestational age, aortic isthmus, aortic valve, pulmonary valve and thymic diameters were available for all 472 fetuses; estimated fetal weight for 468; and fetal sex for 414 (200 female, 214 male), with sex percentages calculated among those determinate cases. No data were imputed.

**Table 2:** Gestational-age-specific diameters (mm, mean ± SD).

GA (Weeks)	*n*	Aortic Isthmus	Aortic Valve	Pulmonary Valve	Thymus
19	22	1.78 ± 0.17	2.41 ± 0.27	3.04 ± 0.33	10.67 ± 1.21
20	132	1.89 ± 0.33	2.70 ± 0.38	3.42 ± 0.43	12.06 ± 1.38
21	153	2.02 ± 0.35	2.95 ± 0.38	3.74 ± 0.47	13.57 ± 1.97
22	97	2.09 ± 0.31	3.18 ± 0.39	4.07 ± 0.51	14.38 ± 1.93
23	47	2.35 ± 0.37	3.47 ± 0.40	4.47 ± 0.49	15.63 ± 2.06
24	21	2.67 ± 0.36	3.91 ± 0.68	5.06 ± 0.82	17.91 ± 2.50

GA, gestational age.

**Table 3:** Bivariate Pearson correlations with aortic isthmus diameter (*n* = 472).

Variable (vs. Aortic Isthmus)	Pearson r	*p* Value
Gestational age	0.512	<0.001
Aortic valve diameter	0.507	<0.001
Pulmonary valve diameter	0.516	<0.001
Thymus transverse diameter	0.397	<0.001

**Table 4:** Three-step hierarchical linear regression of aortic isthmus diameter (*n* = 472).

Predictor	B	SE	β	*p* Value
Model 1 (R^2^ = 0.262; F = 167.1; *p* < 0.001)				
Gestational age	0.159	0.012	0.512	<0.001
Model 2 (R^2^ = 0.322; ΔR^2^ = 0.060; F-change = 20.73; *p* < 0.001)				
Gestational age	0.080	0.017	0.256	<0.001
Aortic valve	0.138	0.049	0.186	0.005
Pulmonary valve	0.109	0.040	0.187	0.006
Model 3 (R^2^ = 0.323; ΔR^2^ = 0.0003; F-change = 0.22; *p* = 0.636)				
Gestational age	0.076	0.019	0.245	<0.001
Aortic valve	0.136	0.049	0.183	0.006
Pulmonary valve	0.107	0.040	0.183	0.008
Thymus	0.004	0.008	0.025	0.636

B, unstandardized coefficient (per week for gestational age, per millimeter for the biometric terms); SE, standard error; β, standardized coefficient. Variance inflation factors ranged from 1.88 to 3.28 in Model 3; the highest variance inflation factor in Model 2 was 3.22.

**Table 5:** Hierarchical models with alternative biometric-derived age indices substituted for the clinical gestational age (*n* = 472).

Size Index	r with AoI	R^2^, Model 1	R^2^, Model 2	R^2^, Model 3	ΔR^2^ (Valve Terms)	AV *p*	PV *p*	Thymus *p*
Gestational age	0.512	0.262	0.322	0.323	0.060	0.005	0.006	0.636
FL-derived age equivalent	0.529	0.279	0.330	0.330	0.050	0.019	0.004	0.916
BPD-derived age equivalent	0.535	0.287	0.339	0.339	0.053	0.006	0.016	0.946
HC-derived age equivalent	0.548	0.301	0.338	0.338	0.037	0.013	0.034	0.730

AoI, aortic isthmus; AV, aortic valve; PV, pulmonary valve; FL, femur length; BPD, biparietal diameter; HC, head circumference. FL-, BPD- and HC-derived ages are the gestational-age equivalents recorded in the clinical reports for the corresponding biometric parameter, expressed in total days. No formal comparison between indices was performed and these R^2^ values do not rank the indices.

**Table 6:** Isthmus-to-valve ratios by completed gestational week (*n* = 472).

GA (Weeks)	*n*	AoI/AV Mean ± SD	AoI/AV P5–P95	AoI/PV Mean ± SD	AoI/PV P5–P95
19	22	0.749 ± 0.110	0.621–0.905	0.594 ± 0.099	0.467–0.714
20	132	0.707 ± 0.135	0.523–0.946	0.557 ± 0.103	0.418–0.712
21	153	0.693 ± 0.128	0.494–0.919	0.546 ± 0.099	0.404–0.758
22	97	0.664 ± 0.106	0.510–0.858	0.519 ± 0.088	0.398–0.667
23	47	0.682 ± 0.116	0.541–0.958	0.529 ± 0.088	0.400–0.662
24	21	0.698 ± 0.131	0.543–0.912	0.535 ± 0.080	0.435–0.641
All	472	0.693 ± 0.125	0.515–0.920	0.544 ± 0.097	0.405–0.714

GA, gestational age; AoI, aortic isthmus; AV, aortic valve; PV, pulmonary valve; P5–P95, empirical 5th to 95th percentile.

**Table 7:** Descriptive equations for aortic isthmus diameter (*n* = 472).

Predictor (x)	Conditional Mean (mm)	Conditional SD (mm)	Valid Range of x	Model R^2^
Gestational age (days)	−1.39005 + 0.022746·x	−0.18339 + 0.003328·x	133–174 days	0.262
FL-derived gestational-age equivalent (days)	−1.07274 + 0.020428·x	−0.00222 + 0.002054·x	132–185 days	0.279

SD, standard deviation; FL, femur length. The conditional standard deviation was obtained from absolute residuals scaled by √(π/2) and modeled on the predictor by iteratively weighted least squares, following Altman [11]; the √(π/2) scaling is already contained in the coefficients shown. The predicted standard deviation is positive throughout the stated ranges (0.259–0.396 mm and 0.269–0.378 mm, respectively).

## Data Availability

The de-identified analytic dataset underlying the reported analyses is provided as Supplementary Dataset S1, and the analysis scripts that reproduce the reported results are provided as Supplementary Files S1 and S2. No data beyond those deposited are publicly available; source clinical records held in the institutional ultrasound archive are not available for sharing because they contain identifiable patient information.

## Supplementary Materials

The following supporting information can be downloaded at: https://www.mdpi.com/article/10.3390/jcm15207798/s1, Supplementary Dataset S1: De-identified minimal analytic dataset (472 records × 13 variables); Supplementary File S1: Python analysis script reproducing the reported analyses; Supplementary File S2: Python script generating Figure 2 and the descriptive equations; Supplementary File S3: Completed STROBE checklist for cohort studies.
